# Supplementary material for: MetaRibo-Seq measures translation in microbiomes
Source: Nat Commun. 2020 Jun 29;11:3268. doi: 10.1038/s41467-020-17081-z (PMC7324362; doi:10.1038/s41467-020-17081-z)
Supplement: Supplementary file 10 — Supplementary Data 7 [file 41467_2020_17081_MOESM10_ESM.zip › File2/Confidence_High_Taxonomy/241708_out.krona.html]

Javascript must be enabled to view this page.

members
magnitude
magnitudeUnassigned
count
unassigned
taxon
rank

241708\_out

4

4
superkingdom
2

4
phylum
976

200643
class
4

4
order
171549

4
family
171552

4
genus
838

165179

SRS012849\_contig\_number\_5746SRS013521\_contig\_number\_10168SRS053356\_contig\_number\_contig-100\_156.275122
3
species

59823

SRS013098\_contig\_number\_contig-100\_1423.292597
1
species
